# Characterization of pig tonsils as niches for the generation of *Streptococcus suis *diversity

**DOI:** 10.1186/s13567-024-01270-5

**Published:** 2024-02-06

**Authors:** Kai Kobayashi, Hiroaki Kubota, Mari Tohya, Megumi Ushikubo, Miki Yamamoto, Tsukasa Ariyoshi, Yumi Uchitani, Morika Mitobe, Rumi Okuno, Ichiro Nakagawa, Tsutomu Sekizaki, Jun Suzuki, Kenji Sadamasu

**Affiliations:** 1 Department of Microbiology, Tokyo Metropolitan Institute of Public Health, Hyakunincho 3-24-1, Shinjuku-ku, Tokyo, 169-0073 Japan; 2 Division of Biomedical Food Research, National Institute of Health Sciences, Tonomachi 3-25-26, Kawasaki-ku, Kawasaki-shi, Kanagawa, 210-9501 Japan; 3 Department of Microbiology and Department of Microbiome Research, Juntendo University School of Medicine, Hongo 2-1-1, Bunkyo-ku, Tokyo, 113-8421 Japan; 4 Shibaura Meat Sanitary Inspection Station, Tokyo Metropolitan Government, Konan 2-7-19, Minato-ku, Tokyo, 108-0075 Japan; 5 Department of Microbiology, Graduate School of Medicine, Kyoto University, Yoshida-Konoe-cho, Sakyo-ku, Kyoto, 606-8501 Japan; 6 Research Center for Food Safety, Graduate School of Agricultural and Life Sciences, The University of Tokyo, Yayoi 1-1-1, Bunkyo-ku, Tokyo, 113-8657 Japan

**Keywords:** *Streptococcus suis*, MLST, clonal complex, *cps* genotype, serotype, virulence-associated markers, porcine endocarditis, tonsil

## Abstract

*Streptococcus suis* is a gram-positive bacterium that causes meningitis, septicemia, endocarditis, and other disorders in pigs and humans. We obtained 42 and 50 *S. suis* isolates from lesions of porcine endocarditis and palatine tonsils, respectively, of clinically healthy pigs in Japan; we then determined their sequence types (STs) by multilocus sequence typing (MLST), *cps* genotypes, serotypes, and presence of classical major virulence-associated marker genes (*mrp*, *epf*, and *sly*). The 42 isolates from endocarditis lesions were assigned to a limited number of STs and clonal complexes (CCs). On the other hand, the 50 isolates from tonsils were diverse in these traits and seemingly in the degree of virulence, suggesting that tonsils can accommodate a variety of *S. suis* isolates. The goeBURST full algorithm using tonsil isolates obtained in this study and those retrieved from the database showed that major CCs as well as many other clusters were composed of isolates originating from different countries, and some of the STs were very similar to each other despite the difference in country of origin. These findings indicate that *S. suis* with not only different but also similar mutations in the genome have survived in tonsils independently across different geographical locations. Therefore, unlike the lesions of endocarditis, the tonsils of pigs seemingly accommodate various *S. suis* lineages. The present study suggests that *S. suis* acquired its diversity by natural mutations during colonization and persistence in the tonsils of pigs.

**Supplementary Information:**

The online version contains supplementary material available at 10.1186/s13567-024-01270-5.

## Introduction

*Streptococcus suis* (*S. suis*), a Gram-positive bacterium, is an important swine pathogen that causes meningitis, septicemia, sudden death, pneumoniae, and arthritis in farmed pigs. In contrast, clinically healthy pigs are often found to carry endocarditis during slaughter [1]. *S. suis* showed affinity to pig oral cavities, as 100% of examined pigs carried *S. suis* in their saliva [2, 3]. *S. suis* may also affect humans and other animals [4]. *S. suis* strains were classified into 35 serotypes (serotypes 1 to 34 and 1/2 which reacts to antisera type 1 and 2) on the basis of the antigenicity of capsular polysaccharide antigens [5, 6]. However, the serotypes 20, 22, 26, 32, 33 and 34 were moved from *S. suis* on the basis of molecular phylogenetic analyses, and the remaining 29 serotypes have so far been officially recognized as *S. suis* [7–9].

The capsule of *S. suis* is encoded by a cluster of capsular polysaccharide synthesis (*cps*) genes located in a single locus of the genome [10]. Typing of the *cps* gene for all serotypes by two-step multiplex PCR has been developed [11]. Conventional serotyping using serum agglutination is time-consuming and it is costly to purchase or prepare all the typing antisera; thus, it is ideal to conduct genotyping by PCR (referred to as *cps* genotyping) in advance, followed by serum agglutination test using expected antisera. From investigations of field isolates of *S. suis*, most isolates from diseased pigs belonged to limited serotypes (serotypes 2, 3, 7, and 9); in particular, the isolates of serotype 2 were highly virulent and most prevalent amongst all serotypes [1, 4, 5, 12].

The capsule of *S. suis* acts as an important virulence factor by escaping from phagocytosis by macrophages and neutrophils of the host defense system [13–15]. Since isogenic un-encapsulated mutants showed a low degree of virulence in pigs and mice, un-encapsulated *S. suis* is believed to be avirulent [16–18]. Furthermore, recent experiments of several serotypes and replacing the total *cps* gene cluster showed that the capsule itself altered the degree of virulence in *S. suis* [19]. In contrast, both encapsulated and un-encapsulated *S. suis* have been found to persist in lesions of porcine endocarditis [9]. Un-encapsulated isolates showed increased ability to adhere to porcine and human platelets and intercellular matrix proteins, to invade to cultured porcine cells, and to form biofilms [20–23]. In addition, the loss of the capsule caused by spontaneous mutations in one or more of *cps* genes possibly occurred during persistent infection in pig bodies [8, 20, 24].

In addition to the capsule, many other potential virulence factors have been described, including muramidase-released protein (MRP, encoded by *mrp*) [25], extracellular factor (EF, encoded by *epf*) [26], and suilysin (SLY, encoded by *sly*) [27]. The precise roles of MRP and EF have not been identified; however, these putative virulence factors have been frequently found to be associated with highly virulent serotype 2 isolates [28, 29]. Although many additional virulence-associated markers have been proposed, the three markers of MRP, EF and SLY still commonly serve as classical virulence markers.

To date, genetic heterogeneity and the phylogenetic diversity of *S. suis* strains have been described using various molecular tools [30–36]. However, multilocus sequence typing (MLST) is currently one of the most valuable tools for examining the population structure and global distribution of *S. suis* [37–42]. Notably, the results of MLST analysis can be visualized by a bioinformatic tool, goeBURST [43]. *S. suis* serotype 2 can be classified by MLST into more than 10 sequence types (STs), among which some closely related STs can be grouped as ST clonal complexes (CCs) by goeBURST. For example, strains of *S. suis* serotype 2 with high virulence were classified into ST1 or ST7, which belonged to CC1. In contrast, some low or non-virulent strains were allotted to many other STs and could not be assigned to a certain CC [38, 40], indicating that there is higher diversity of low or non-virulent strains than highly virulent strains.

**Figure 1:**
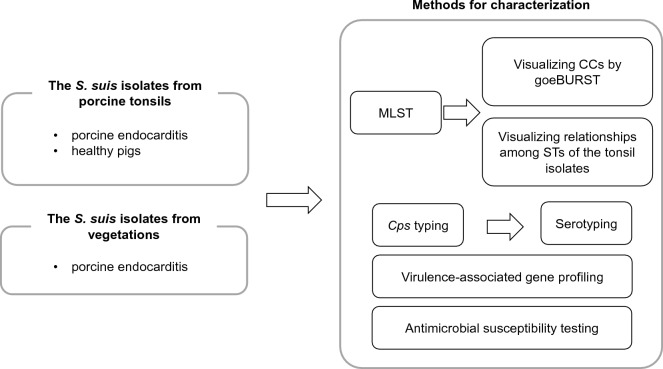
**The scheme of the present study design**

Through the use of the typing tools described above, many studies have been performed to elucidate the determinant for the virulence of *S. suis*, utilizing isolates from both diseased and healthy pigs as well as human cases [28, 33, 44–47]. On the contrary, studies using comparison or population analysis of the low or non-virulent isolates of *S. suis* are rare. In the present study, we focused on low or non-virulent isolates of *S. suis* from healthy pigs to examine their population structure and relationship in order to clarify *S. suis* diversity. The total design of this study is depicted in Figure 1.

## Materials and methods

### Isolation and identification of *S. suis*

Organ samples were collected from 110 pigs shipped to a meat sanitary inspection station in Tokyo from 22 farms in Tohoku and Kanto areas of Japan between 2015 and 2021. All the pigs were considered clinically healthy before slaughter; however, at the time of inspection, 50 pigs of marketing ages (approximately 6 months of age), reproductive ages (2 to 3 years of age), and age unknown were condemned because of endocarditis. The rest of 60 pigs were marketing age. We defined the 60 pigs as healthy pigs in the present study. Samples of heart valve vegetations from 42 pigs and of palatine tonsils from 68 pigs (8 with endocarditis and 60 healthy) were stamped onto either Trypticase soy agar (Becton, Dickinson and Company, Sparks, MD, USA) supplemented with 5% horse blood (TSA-HB) or Columbia agar (OXOID, Kanto Chemical Co., Tokyo, Japan) supplemented with Streptococcus selective supplement (Thermo Fisher Scientific, Waltham, USA) and 5% horse blood (COBA), and then incubated at 37 °C for 48 h in air, and if necessary, passaged to TSA-HB or COBA and incubated for 24 h in air or air plus 5% CO_2_, respectively. Colonies grown on the agar with α-hemolysis were selected and screened by Gram staining, catalase test, and rapid identification kit (Rapid ID32 Strep API, BioMérieux, Marcy-l’Étoile, France). The suspected isolates were confirmed to be *S. suis* by species-specific PCR for *S. suis* (*recN* PCR) [48]. The *S. suis* isolates were frozen in skim milk (Becton Dickinson) at −80 °C until use.

### DNA extraction and PCR

The *S. suis* isolates grown on TSA-HB at 37 °C for 24 h in air plus 5% CO_2_ were used for bacterial DNA extraction following the alkaline boiling method [49]. Briefly, the bacteria were suspended in 25 mM NaOH, heated at 95 °C for 10 min, and then neutralized by adding an equal volume of 80 mM Tris–HCl (pH 8.0), followed by centrifugation at 13 000 × *g* for 3 min. The supernatants were used for PCR. AmpliTaq Gold 360 Master Mix (Thermo Fisher Scientific) was used for PCR amplification according to the manufacturer’s instructions.

### MLST

As described previously, MLST was performed by direct sequencing of seven PCR-amplified house-keeping genes [37]. The allele numbers and sequence type (ST) of the isolates were determined by comparing their sequences with those in the PubMLST database [63] accessed in May 2023. Novel alleles and STs were assigned by submitting the respective data to the database administrator. The goeBURST (v1.2) algorithm from Phyloviz 2.0 software [50] was used to visualize CCs by creating an MLST-based minimal spanning tree (MST) with all the isolates obtained in this study and all strains retrieved from the PubMLST database (2273 STs on May 11, 2023) at the triple-locus variant level (TLV). CCs were composed of STs with at least 6 identical alleles, except that ST117, ST1528, and ST1529 were included in CCs as they were double-locus variants (DLVs) or TLVs of major CCs. Among the STs that could not be assigned to any CC, those that differed in 4 or more loci from their closest counterparts were defined as singletons. For visualizing relationships among STs of the tonsil isolates, the 50 isolates obtained in this study and 559 isolates with tonsil origin noted in the database were linked by the goeBURST full algorithm in Phyloviz 2.0. From the database, MLST profiles of 9 or more deposited from the same country were selected for this analysis and the differences in countries were color-coded.

### Capsular polysaccharide synthesis (*cps*) gene profiles and serotyping

Genotypes of *cps* genes were determined by the 2-step multiplex PCR assay previously described [11]. If the PCR fragments could not be obtained or the sizes of the fragments were different from those described in the original study, such cases were classified as untypeable and were not subject to serum agglutinations. A mismatch amplification mutation assay-PCR [51] was used to distinguish type 1 and 14, and type 2 and 1/2. If the *cps* genotypes could be determined, the serotypes were determined using an expected type of commercially available antisera by either co-agglutination tests or slide agglutinations [20]. In cases where the positive agglutination was not observed, such isolates were defined as untypeable.

### Virulence-associated gene profiling

Three virulence-associated genes, muramidase-released protein gene (*mrp*), extracellular factor gene (*epf*), and suilysin gene (*sly*), were examined by multiplex PCR using primers as previously described [28]. Furthermore, conventional PCRs were also used for the differentiation of variants of *mrp* [28] and *epf* [52]. Variants of MRP were expressed as *mrp*^S^ (747 bp), *mrp* (1148 bp), mrp* (1556 bp), and mrp*** (2400 bp). A large-size variant of *epf* was expressed as epf*.

### Antimicrobial susceptibility testing

The minimum inhibitory concentrations (MICs) of the tested antibiotics were determined using a broth microdilution method with a commercially available kit (Eiken Chemical, Tokyo, Japan) for eight antimicrobial agents (penicillin [PCG], ampicillin [ABPC], cefepime [CFPM], ceftriaxone [CTRX], azithromycin [AZM], clindamycin [CLDM], clarithromycin [CAM], and levofloxacin [LVFX]) and Etest (BioMérieux) for one antimicrobial agent (tetracycline [TC]). The MIC breakpoints were taken from the Clinical and Laboratory Standard Institute (CLSI) criteria in M100-ED33 for the *Streptococcus* spp. viridans group [53]. Fisher’s exact test (two-tailed) was used to test for statistical significance. Statistical significance was set at *p* < 0.05.

## Results

### Characterization of *S. suis* isolates by MLST and goeBURST

**Figure 2:**
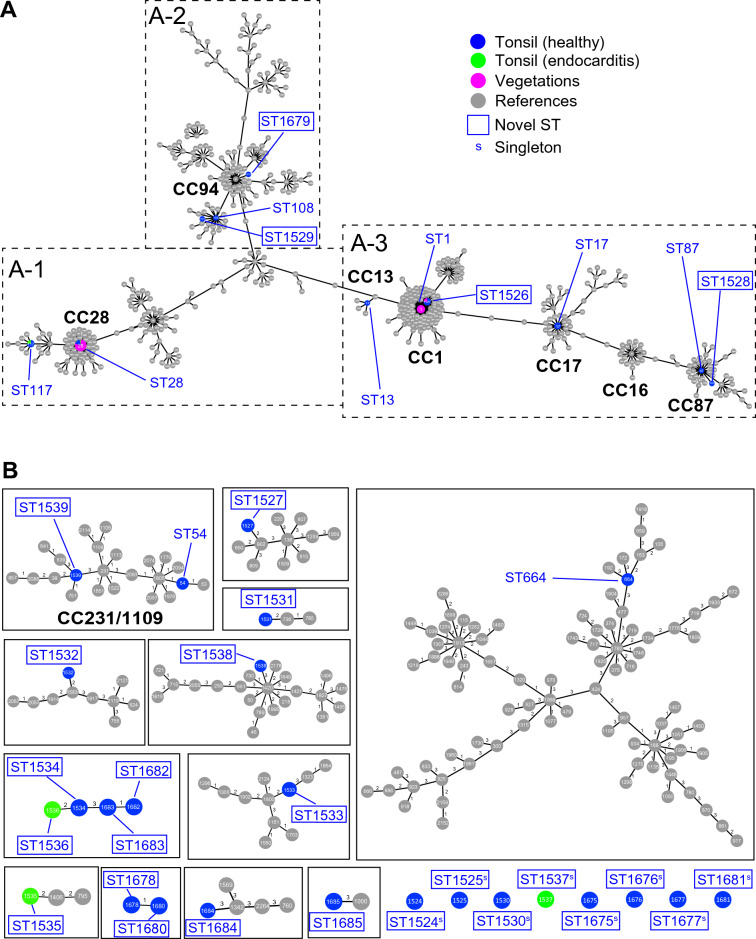
**goeBURST analysis using all *S. suis* isolates described in this study and all strains retrieved from the PubMLST database (2273 STs) at the triple-locus variant level visualizing clonal complexes.** **A-1**–**A-3** Major clonal complexes related to each other. ST117 is related to ST28 via ST27 (i.e., a double-locus variant of ST28) and is included in CC28. ST1528 is linked to ST87 at the double-locus variant level, and is included in CC87. ST1529 is related to ST94 via ST108 (i.e., a triple-locus variant of ST94) and is included in CC94. **B** Independent CCs and Singletons

Forty-two *S. suis* isolates were identified from all the vegetation samples examined, and 5 and 45 *S. suis* isolates were identified from the palatine tonsils of 8 pigs with endocarditis and that of 60 healthy pigs, respectively. The details of the isolates are listed in Additional file 1. A total of 92 isolates were assigned to 36 STs by MLST analysis (Table 1). Among them, 31 isolates were assigned to 27 novel STs (ST1524-ST1539 and ST1675-ST1685). The goeBURST algorithm (v1.2) at the TLV level showed that the 42 isolates from endocarditis lesions were assigned to either CC1 (ST1 and ST1526) or CC28 (ST28) (Table 1, Figure 2A (panels A-1 and A-3)]. On the contrary, among the 50 isolates from palatine tonsils, three and twelve isolates were assigned to CC1 and CC28, respectively, nine isolates were assigned to various previously identified CCs, and among them, six isolates belonged to novel STs (Table 1, Figures 2A and B). The remaining 26 isolates from palatine tonsils were not assigned to any CCs; two isolates were assigned to previously identified STs (ST54 and ST664), and 24 isolates belonged to novel STs (Table 1).

**Table 1:** **MLST analysis, *cps*** **type and profiles of virulence-associated genes of the 92** ***S. suis*** **isolates**

Clonal complex (CC)	Sequence type (ST)	No. of isolates	*cps* type	*mrp*	*epf*	*sly*
*S. suis* isolates from vegetations of porcine endocarditis						
1	1	7	2	+, −	+	+
	1526^a^	1	2	−	+	+
28	28	34	2, UT	+	−	−
*S. suis* isolates from porcine tonsils						
1	1526^a^	3	2	−	+	+
28	28	8	2	+	−	−
	117	4	3	***	−	−
13	13	1	14	−	−	+
17	17	3	4	S	*	+
87	87	1	8	−	−	+
	1528^a^	1	8	−	−	+
94	108	1	5	+	−	+
	1529^a^	1	7	+	−	+
	1679^a^	1	4	+	−	+
Not assigned^b^	54	1	3	−	−	+
	664	1	16	−	−	−
	1527^a^	1	9	−	−	−
	1531^a^	1	UT	*	−	+
	1532^a^	1	31	−	−	−
	1533^a^	2	UT	−	−	+
	1534^a^	1	UT	***	−	+
	1535^a^	1	15	***	−	+
	1536^a^	1	11	*	*	+
	1538^a^	1	16	−	−	−
	1539^a^	1	UT	−	*	+
	1678^a^	1	12	***	−	+
	1680^a^	1	12	*	−	+
	1682^a^	1	UT	+	−	+
	1683^a^	1	UT	*	−	+
	1684^a^	1	31	−	−	−
	1685^a^	1	10	−	−	−
Singleton^c^	1524^a^	1	16	−	−	−
	1525^a^	1	UT	−	−	−
	1530^a^	1	UT	−	−	−
	1537^a^	1	31	−	−	−
	1675^a^	1	31	−	−	−
	1676^a^	1	5	−	−	−
	1677^a^	1	11	*	−	−
	1681^a^	1	31	−	−	−

Variants of *mrp* are shown as *** (2400 bp), * (1556 bp), and S (747 bp). A large-size variant of *epf* is shown as *.

^a^Novel STs.

^b^Single- to triple-locus variants of other STs; however, clonal complex could not be assigned.

^c^More than 4 loci were different from other STs.

On the other hand, among the 27 novel STs, four (ST1526, ST1528, ST1529, and ST1679) were assigned to previously described CCs (Table 1, Figure 2A (panels A-2 and A-3)). Fifteen novel STs were linked to previously identified or novel STs at least at the TLV level (Table 1, Figure 2B). Remarkably, two small clusters, one comprising ST1536, ST1534, ST1683, and ST1682, and the other comprising ST1678 and ST1680, consisted of only novel STs (Figure 2B). Notably, ST54 and ST1539 were close to STs that were previously assigned to CC231/1109 (Figure 2B) [40]. However, eight remaining novel STs were considered to be singletons. The closest STs to these singletons were quadruple-locus variants or quintuple-locus variants (Additional file 2).

### Relationship among the 50 tonsil isolates and other previously identified tonsil isolates

**Figure 3:**
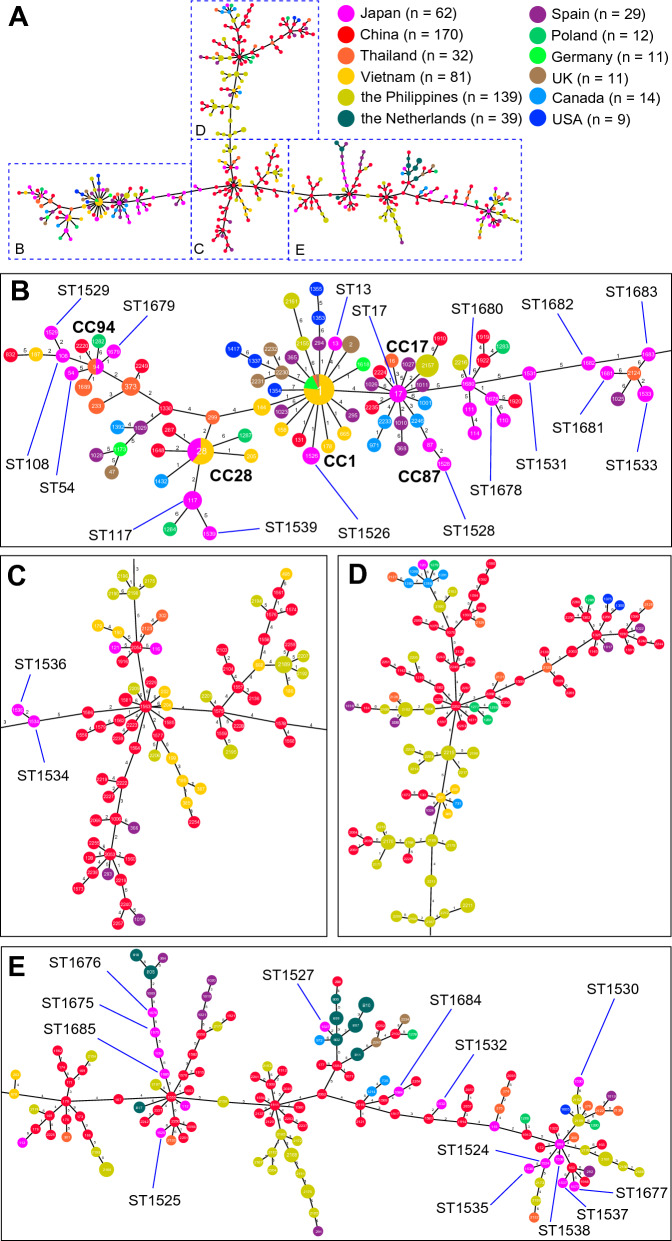
**MLST-based minimal spanning tree of 50 *S. suis* isolates from tonsils described in this study and 559 isolates of tonsil origins in the database.** The tree was calculated using the goeBURST full algorithm. Numbers within the nodes indicate the corresponding sequence type. Node colors refer to the origin of countries as represented in the remarks. Numbers on lines indicate locus variants between nodes

MLST-based MST calculated by the goeBURST full algorithm in Phyloviz 2.0 could link all the examined tonsil isolates (Figure 3). Except for two STs, ST1354 and ST2230, which were linked to ST1 at the septuple-locus variant level (Figure 3B), all the STs showed at least one locus that was identical to their counterparts. The STs of tonsil isolates were clustered with not only Japanese isolates but also those from many other countries. In the CC1, CC17, CC28, and CC94 clusters, the representative STs were surrounded by other STs with isolates originating from other countries (Figure 3B). In addition to the clusters involved in and close to CCs, isolates from different countries formed many clusters. Although most STs differed in more than 3 loci from their counterparts, some were single-locus variants (SLVs) or DLVs of other STs despite the difference in country of origin (Additional file 3).

### Characterization of *S. suis* isolates by *cps* typing and serotyping

The *cps* types and serotypes are summarized in Table 2. *Cps* typing of the 42 isolates from vegetations of endocarditis identified only *cps*2 except for an untypeable isolate. Among them, 20 isolates were serotype 2, and the expected antiserum did not agglutinate the remaining 21 isolates and were untypeable serotypes. Among the 50 isolates from tonsils, nine were *cps* untypeable. The remaining 41 isolates were assigned to 14 different *cps* types. Three of them, belonging to CC1, were *cps*2. Among them, two were serotype 2, and one was untypeable serotype. The eight and four isolates belonging to CC28 were *cps*2 and *cps*3, respectively; the former was serotype 2 and the later was serotype 3. Among the remaining 35 isolates, *cps* types could be determined for 26 isolates and serotypes for 23 isolates (Table 2). Most *cps* types harbored one or two STs, whereas *cps*16 and *cps*31 harbored three and five STs, respectively, in which one and three were singletons. *Cps*2 were commonly found in the isolates from both vegetations of endocarditis and tonsils, and STs in the isolates from vegetations and tonsils were similar. On the other hand, other *cps* types were found only in the isolates from tonsils (Table 2).

**Table 2:** ***Cps*** **types and serotypes of 92** ***S. suis*** **isolates, and their clonal complexes (CCs) and sequence types (STs)**

*cps* type	No. of isolates belonging to serotypes below														CC	ST
	2	3	4	5	7	8	9	10	11	12	15	16	31	UT		
*S. suis* isolates from vegetations of porcine endocarditis																
2	5													3	1	1, 1526
	15													18	28	28
UT														1^a^	28	28
*S. suis* isolates from porcine tonsils																
14														1	13	13
2	2													1	1	1526
	8														28	28
3		4													28	117
		1													NA^b^	54
4			3												17	17
			1												94	1679
5				1											94	108
				1											NA	1676^c^
7					1										94	1529
8						2									87	87, 1528
9							1								NA	1527
10								1							NA	1685
11									1					1	NA	1536, 1677^c^
12										2					NA	1678, 1680
15											1				NA	1535
16												3			NA	664, 1524^c^, 1538
31													4	1	NA	1532, 1537^c^, 1675^c^, 1681^c^, 1684
UT														9^a^	NA	1525^c^, 1530^c^, 1531, 1533, 1534, 1539, 1682, 1683

^a^Not determined.

^b^No clonal complex could be assigned.

^c^Singleton.

### Characterization of *S. suis* isolates by virulence-associated gene profiles

Virulence-associated gene profiles are summarized in Table 1. The 42 isolates from vegetations of endocarditis showed only three patterns of the genotypes, i.e., *mrp*+*epf*+*sly*+, *mrp−* *epf*+*sly*+, and, *mrp*+*epf*− *sly*−. On the other hand, the 50 isolates from tonsils showed various genotypes. The same genotypes seen in the isolates from vegetations were only found in three isolates of CC1 (ST1526, *cps*2) and eight isolates of CC28 (ST28, *cps*2). Furthermore, the typical profile of ST1 (*cps*2), an *mrp*+*epf*+*sly*+ genotype, was not found among the isolates from tonsils, and the 13 isolates of unassigned CC, including 12 novel STs and seven singletons (ST664, ST1524, ST1525, ST1527, ST1530, ST1532, ST1537, ST1538, ST1675, ST1676, ST1681, ST1684 and ST1685 corresponding to the following *cps* types, *cps*5, *cps*9, *cps*10, *cps*16, *cps*31 and *cps* untypeable), showed a triple negative genotype, *mrp−* *epf*− *sly*− (Table 1).

### Antimicrobial susceptibility

According to the measured MICs and the CLSI criteria, 67.4%, 67.4%, 72.8%, 77.2%, and 2.2% of isolates were resistant to AZM, CAM, CLDM, TC, and PCG, respectively (Table 3). No significant difference in susceptibility to any antimicrobial agents was observed between isolates from tonsils and vegetations of endocarditis (*p* > 0.05). Furthermore, 60 of all 92 isolates (65.2%) showed multidrug resistance to the four agents AZM, CAM, CLDM, and TC (Additional file 1).

**Table 3:** **Number of isolates and resistance rates (in brackets) of each antimicrobial agents by origin and sequence types (STs)**

Agent^*^	Total *n* = 92	Origin		ST	
		Tonsils *n* = 50	Vegetations *n* = 42	ST28 *n* = 42	Other STs *n* = 50
AZM	62 (67.4%)	30 (60.0%)	32 (76.2%)	29 (69.0%)	33 (66.0%)
CAM	62 (67.4%)	30 (60.0%)	32 (76.2%)	29 (69.0%)	33 (66.0%)
CLDM	67 (72.8%)	35 (70.0%)	32 (76.2%)	29 (69.0%)	38 (76.0%)
TC	71 (77.2%)	38 (76.0%)	33 (78.6%)	32 (76.2%)	39 (78.0%)
PCG	2 (2.2%)	2 (4.0%)	0 (0.0%)	0 (0.0%)	2 (4.0%)

^*^The minimum inhibitory concentrations were tested for penicillin (PCG), ampicillin (ABPC), cefepime (CFPM), ceftriaxone (CTRX), azithromycin (AZM), clarithromycin (CAM), clindamycin (CLDM), levofloxacin (LVFX), and tetracycline (TC), and breakpoints were taken from the Clinical and Laboratory Standard Institute (CLSI) criteria in M100-ED33 for the *Streptococcus* spp. viridans group (CLSI, 2023). There was no isolate resistant for ABPC, CFPM, CTRX, and LVFX.

## Discussion

The MLST analyses of *S. suis* isolated from diseased pigs and clinically healthy pigs have been described in many previous studies and there have been many efforts to discriminate the highly virulent *S. suis* isolates from low or non-virulent isolates [40, 46, 47, 54–58]. Although highly virulent strains that may affect mortality of nursery piglets have been spotlighted, low or non-virulent strains that occupy the majority of *S. suis* may play a part of the whole ecosystem. This implies that the low or non-virulent stains may affect the healthy status of not only nursery piglets but also pigs of other ages. In the present study, we isolated and examined *S. suis* from clinically healthy pigs to elucidate the diversity of and relationships among presumably low virulent *S. suis* isolates using MLST analysis. In a previous study, we showed that prevalence of *S. suis* in Japan was almost similar from north to south parts of Japan [2], this suggests that the results obtained in this study can be extrapolated to other areas because the samples were collected in the meat inspection center in Tokyo, the biggest consumption area in Japan. As has been previously described [8, 9, 20], the isolates from endocarditis lesions belonged to a limited number of CCs, CC1 and CC28, which are reported to involve highly virulent isolates [29, 37–39, 46, 47, 56]. On the other hand, fifteen isolates from tonsils belonged to CC1 or CC28, consistent with previous studies reporting that potentially hazardous *S. suis* persists in asymptomatic pigs [59, 60]. To date, in addition to CC1 and CC28, CC16, CC17, CC20, CC25, CC94, CC104, CC233/379, and CC221/234 have been reported to involve highly virulent or potentially hazardous *S. suis* [56]. ST28 was predominant in the isolates from endocarditis and found most in those from tonsils. However, as far as the typing of MLST, serotypes, and virulence-associated genes, we could not discriminate between the ST28 isolates from endocarditis and those from tonsils. On the contrary, in the present study, the isolates from tonsils showed extensive ST diversity; in particular, 30 of the 50 isolates from tonsils were assigned to 27 novel STs. Four isolates belonging to novel STs (ST1526, ST1528, ST1529, and ST1679) were assigned to previously described CCs that may involve potentially hazardous *S. suis*. In particular, ST1526 is SLV of ST1 and was found in isolates from both tonsils and endocarditis vegetations, suggesting that the isolates belonging to ST1526 were diverged from ST1 and were virulent. However, the remaining 26 isolates belonging to novel STs and two isolates belonging to previously described STs (ST54 and ST664) could not be assigned to any CCs or were considered singletons (Table 1). Although some of these isolates showed relationships to previously described STs or other novel STs at least at the TLV level (Figure 2B), these STs did not seem to involve highly virulent *S. suis*. Furthermore, eight singletons showed no close relationships to other STs (Additional file 2), indicating that the isolates belonging to these novel STs were low or non-virulent *S. suis*. These observations suggest that tonsils can accommodate potentially hazardous *S. suis* and permit a variety of low or non-virulent *S. suis* strains to persistently colonize in this niche by protecting them from the host immune system.

To characterize the potential of tonsils as niches accommodating low or non-virulent *S. suis*, we made a schema of an MLST-based MST calculated by the goeBURST full algorithm using *S. suis* isolates from tonsils of healthy pigs obtained in this study and those retrieved from the database (Figure 3). Since the algorithm was able to connect all the STs even though there are differences in 7 loci from other STs, with only two exceptions, all the examined STs were connected to form a large tree with at least the sextuple-locus variant level (Figure 3). Although the number of the isolates from each country was different, many of the clusters that appeared in the MST were composed of STs whose isolates originated from different countries. Furthermore, many STs did not belong to major CCs, that is, most STs seemed to contain low or non-virulent *S. suis* strains. Notably, although most of the STs were linked at lower than TLV levels, some STs whose isolates originated from different countries were connected at SLV or DLV levels (Additional file 3), indicating that similar mutations affecting the ST have occurred across different countries. Imports of live pigs into Asian countries from either the EU or North American countries for the purpose of breeding take place frequently. It is difficult to determine whether the international trading of pigs shaped the MST clusters of isolates from different countries or whether a selective pressure under the same environment in the tonsils resulted in similar progenies surviving in such environment and forming the clusters in the MST. In the case of humans, frequent and long-distance moving by globalization can rapidly and globally spread the same clone of pathogens, as exemplified by a lineage 4 of *Mycobacterium tuberculosis* [61]. In contrast to humans, pigs in farms are not frequently transported between different countries and regions. Therefore, such a high degree of diversity found in tonsil isolates and survivals of the same or related STs may be caused by natural mutations followed by selective pressures that occurred during multiplications of the bacteria in each country, presumably in pig bodies, rather than carryover of *S. suis* from exporting countries. Although the ST28 strains, which were predominant in this study, could be imported by the international trading from other ST28 endemic countries (e.g., North America [55]), these strains have already been established and circulating in Japan [59].

All the isolates except 1 (untypeable) from endocarditis lesions were *cps* type 2, as reported previously [9, 20, 59]. Among them, 21 isolates did not show agglutination against serotype 2 antiserum, suggesting that these isolates were un-encapsulated, as observed previously [8, 9, 20] (Table 2). On the other hand, only 11 isolates from tonsils were *cps* type 2, of which 10 isolates were serotype 2. Although we do not have any experimental evidence, the one isolate that did not show positive agglutination seemed to be un-encapsulated. Interestingly, one isolate each in *cps* types 1, 11, and 31 did not show positive agglutination against expected types of antisera, suggesting that these isolates also seemed to be un-encapsulated. The un-encapsulated *S. suis* is believed to be avirulent because of weakness against phagocytosis by the host defense systems [1, 13, 14, 16, 17]. However, un-encapsulated *S. suis* exhibits increased levels of adhesion to host cells and biofilm formation that may confer upon the bacteria an ability to persistently colonize and resist clearance by host immune systems [14, 20, 22]. Assuming that the isolates that did not show agglutination were un-encapsulated, they may have persisted in tonsils through the same mechanism of persistence in endocarditis lesions. Although we cannot determine the degree of virulence on the basis of the serotypes, the serotypes in conjunction with STs suggest that, in addition to un-encapsulated isolates, most of the isolates from tonsils seemed to be low or non-virulent.

The types of virulence-associated genes varied among previous reports. However, the major three virulence-associated genes examined in the present study were commonly appeared in those studies [38, 40, 59] and easily compare the results among such reports. The isolates from endocarditis lesions showed typical patterns of virulence-associated marker genotypes. On the other hand, only a few isolates from the tonsils showed the typical patterns above; in particular, there was no isolates that showed *mrp*+*epf*+*sly*+, which was frequently found in highly virulent ST1 strains [29, 38]. The fact that various patterns of virulence-associated genes were found and the most frequently found pattern was *mrp*− *epf*− *sly*− as well as the findings on MLST and serotypes strongly suggest that most of the isolates from tonsils were low or non-virulent.

The proportions of resistance to macrolides (AZM and CAM), lincomycins (CLDM), and tetracyclines (TC) were high (approximately 70%) regardless of the origins, with the majority of all isolates presenting multidrug resistance to these four agents. Compared to previous investigations in the Tokai region of Japan, where antimicrobial resistances to CAM, CLDM, and TC were 56.1%, 65.8%, and 80.7%, respectively [62], our results showed similar but higher resistance rates for macrolides and lincomycins.

On the basis of MLST, *cps* genotypes, serotypes, and virulence-associated marker genotypes, the isolates from endocarditis lesions showed similar patterns, indicating that these isolates were highly clonal. On the other hand, the isolates from tonsils showed an extensively high degree of diversity in all the features examined, and most of them seemed to be low or non-virulent; moreover, a variety of low or non-virulent isolates could, in fact, persist in tonsils of pigs across many countries.

Characteristics of endocarditis and tonsils isolates summarized in Table 4 clearly showed their difference, indicating the degree of virulence was apparently different between them.

**Table 4:** **Comparison of characteristics between the isolates from vegetations of endocarditis and tonsils**

Characteristics	Vegetations	Tonsils
CC/ST	Limited	Various
*Cps* type	Limited	Various
Serotype	Limited	Various
Virulence-associated gene	Limited patterns	Various patterns
Resistant antimicrobial agents (The resistance rates > 50%)	AZM, CAM, CLDM, TC	AZM, CAM, CLDM, TC

Contrary to the isolates from endocarditis lesions, which were highly clonal, the isolates from the tonsils of healthy pigs were extensively diverse in MLST analyses, *cps* gene types, serotyping, and virulence-associated marker genotypes. Although most of the isolates from the tonsils seemed to be low or non-virulent, such isolates may colonize and persist in the tonsils. A variety of STs found in the isolates from tonsils suggests that many mutations in the genome have occurred. Some of the STs with isolates obtained in different countries showed a close relationship. Such mutations occurred randomly and incidentally; however, in vivo conditions in tonsils may affect the survival of such mutants; as a result, STs that showed a close relationship with each other could be found in isolates that originated in different countries. Although it is generally difficult to determine whether an isolate is virulent or low or non-virulent, our applied method comparing MLST/serotypes/virulent-associated genes with previous reports can be useful to predict virulent, low or non-virulent strains. Taken all together, the present study suggests that *S. suis* acquired its diversity through natural mutations during colonization and persistence in pig tonsils.

### Supplementary Information

**Additional file 1. Ninety-two *S. suis* isolates obtained in this study and their characteristics.**

**Additional file 2. Eight novel sequence types (STs) assigned to singletons and their relationships to other STs.**

**Additional file 3. STs that were Single-locus Variants (SLVs) or Double-locus variants (DLVs) of other STs originating from different countries.**

## Data Availability

All data relevant to the study are included in the article or enclosed as an additional file.

## Abbreviations

*S. suis*: *Streptococcus suis*
*cps*: capsular polysaccharide synthesis gene
MLST: multilocus sequence typing
MST: minimal spanning tree
ST: sequence type
*mrp*: muramidase-released protein gene
*epf*: extracellular factor gene
*sly*: suilysin gene
