# Supplementary material for: Characterization of pig tonsils as niches for the generation of Streptococcus suis diversity
Source: Vet Res. 2024 Feb 6;55:17. doi: 10.1186/s13567-024-01270-5 (PMC10848530; doi:10.1186/s13567-024-01270-5)
Supplement: Supplementary file 1 — Additional file 1. Ninety-two S. suis isolates obtained in this study and their characteristics. [file 13567_2024_1270_MOESM1_ESM.docx]

**Additional file 1.** **Ninety-two *S. suis* isolates obtained in this study and their characteristics.**

| Isolate ID | Age of host pig | Sequence type (ST) | Clonal complex (CC) | *cps* type | Serotype | Virulence-associated gene profile | Resistant agent |
| --- | --- | --- | --- | --- | --- | --- | --- |
| *S. suis* isolates from vegetations of porcine endocarditis | | | | | | | |
| 19SS15 | marketing | ST1 | CC1 | 2 | 2 | *mrp* ^+^ *epf* ^+^ *sly* ^+^ | AZM, CAM, CLDM, TC |
| 20SS45 | marketing | ST1 | CC1 | 2 | 2 | *mrp* ^+^ *epf* ^+^ *sly* ^+^ | TC |
| 19SS11 | marketing | ST1 | CC1 | 2 | 2 | *mrp* ^-^ *epf* ^+^ *sly* ^+^ | AZM, CAM, CLDM, TC |
| 19SS24 | marketing | ST1 | CC1 | 2 | 2 | *mrp* ^-^ *epf* ^+^ *sly* ^+^ | AZM, CAM, CLDM, TC |
| 19SS04 | marketing | ST1 | CC1 | 2 | UT | *mrp* ^-^ *epf* ^+^ *sly* ^+^ | AZM, CAM, CLDM, TC |
| 19SS32 | reproductive | ST1 | CC1 | 2 | UT | *mrp* ^-^ *epf* ^+^ *sly* ^+^ | AZM, CAM, CLDM, TC |
| 20SS32 | marketing | ST1 | CC1 | 2 | UT | *mrp* ^+^ *epf* ^+^ *sly* ^+^ | AZM, CAM, CLDM, TC |
| 20SS38 | marketing | ST1526 (SLV of ST1) | CC1 | 2 | 2 | *mrp* ^-^ *epf* ^+^ *sly* ^+^ | AZM, CAM, CLDM, TC |
| 19SS01 | marketing | ST28 | CC28 | 2 | 2 | *mrp* ^+^ *epf* ^-^ *sly* ^-^ | AZM, CAM, CLDM, TC |
| 19SS03 | marketing | ST28 | CC28 | 2 | UT | *mrp* ^+^ *epf* ^-^ *sly* ^-^ | AZM, CAM, CLDM, TC |
| 19SS05 | marketing | ST28 | CC28 | 2 | 2 | *mrp* ^+^ *epf* ^-^ *sly* ^-^ | AZM, CAM, CLDM, TC |
| 19SS06 | marketing | ST28 | CC28 | 2 | UT | *mrp* ^+^ *epf* ^-^ *sly* ^-^ | No resistance |
| 19SS07 | marketing | ST28 | CC28 | 2 | 2 | *mrp* ^+^ *epf* ^-^ *sly* ^-^ | No resistance |
| 19SS08 | marketing | ST28 | CC28 | 2 | 2 | *mrp* ^+^ *epf* ^-^ *sly* ^-^ | AZM, CAM, CLDM, TC |
| 19SS09 | unknown | ST28 | CC28 | 2 | UT | *mrp* ^+^ *epf* ^-^ *sly* ^-^ | AZM, CAM, CLDM, TC |
| 19SS10 | marketing | ST28 | CC28 | 2 | UT | *mrp* ^+^ *epf* ^-^ *sly* ^-^ | AZM, CAM, CLDM, TC |
| 19SS12 | marketing | ST28 | CC28 | 2 | UT | *mrp* ^+^ *epf* ^-^ *sly* ^-^ | AZM, CAM, CLDM, TC |
| 19SS13 | marketing | ST28 | CC28 | 2 | UT | *mrp* ^+^ *epf* ^-^ *sly* ^-^ | AZM, CAM, CLDM, TC |
| 19SS14 | marketing | ST28 | CC28 | 2 | 2 | *mrp* ^+^ *epf* ^-^ *sly* ^-^ | AZM, CAM, CLDM, TC |
| 19SS16 | marketing | ST28 | CC28 | 2 | 2 | *mrp* ^+^ *epf* ^-^ *sly* ^-^ | AZM, CAM, CLDM, TC |
| 19SS17 | marketing | ST28 | CC28 | UT | UT | *mrp* ^+^ *epf* ^-^ *sly* ^-^ | AZM, CAM, CLDM, TC |
| 19SS18 | marketing | ST28 | CC28 | 2 | UT | *mrp* ^+^ *epf* ^-^ *sly* ^-^ | AZM, CAM, CLDM, TC |
| 19SS19 | marketing | ST28 | CC28 | 2 | 2 | *mrp* ^+^ *epf* ^-^ *sly* ^-^ | AZM, CAM, CLDM, TC |
| 19SS20 | marketing | ST28 | CC28 | 2 | 2 | *mrp* ^+^ *epf* ^-^ *sly* ^-^ | AZM, CAM, CLDM, TC |
| 19SS21 | marketing | ST28 | CC28 | 2 | UT | *mrp* ^+^ *epf* ^-^ *sly* ^-^ | AZM, CAM, CLDM, TC |
| 19SS22 | marketing | ST28 | CC28 | 2 | UT | *mrp* ^+^ *epf* ^-^ *sly* ^-^ | AZM, CAM, CLDM, TC |
| 19SS23 | marketing | ST28 | CC28 | 2 | UT | *mrp* ^+^ *epf* ^-^ *sly* ^-^ | AZM, CAM, CLDM, TC |
| 19SS25 | marketing | ST28 | CC28 | 2 | 2 | *mrp* ^+^ *epf* ^-^ *sly* ^-^ | AZM, CAM, CLDM, TC |
| 19SS26 | marketing | ST28 | CC28 | 2 | 2 | *mrp* ^+^ *epf* ^-^ *sly* ^-^ | No resistance |
| 19SS27 | marketing | ST28 | CC28 | 2 | 2 | *mrp* ^+^ *epf* ^-^ *sly* ^-^ | No resistance |
| 19SS28 | marketing | ST28 | CC28 | 2 | 2 | *mrp* ^+^ *epf* ^-^ *sly* ^-^ | AZM, CAM, CLDM, TC |
| 19SS29 | marketing | ST28 | CC28 | 2 | UT | *mrp* ^+^ *epf* ^-^ *sly* ^-^ | No resistance |
| 19SS30 | marketing | ST28 | CC28 | 2 | UT | *mrp* ^+^ *epf* ^-^ *sly* ^-^ | No resistance |
| 19SS31 | marketing | ST28 | CC28 | 2 | UT | *mrp* ^+^ *epf* ^-^ *sly* ^-^ | No resistance |
| 20SS36 | marketing | ST28 | CC28 | 2 | UT | *mrp* ^+^ *epf* ^-^ *sly* ^-^ | AZM, CAM, CLDM, TC |
| 20SS37 | marketing | ST28 | CC28 | 2 | UT | *mrp* ^+^ *epf* ^-^ *sly* ^-^ | No resistance |
| 20SS39 | marketing | ST28 | CC28 | 2 | UT | *mrp* ^+^ *epf* ^-^ *sly* ^-^ | No resistance |
| 20SS40 | marketing | ST28 | CC28 | 2 | 2 | *mrp* ^+^ *epf* ^-^ *sly* ^-^ | AZM, CAM, CLDM, TC |
| 20SS41 | marketing | ST28 | CC28 | 2 | 2 | *mrp* ^+^ *epf* ^-^ *sly* ^-^ | AZM, CAM, CLDM, TC |
| 20SS42 | marketing | ST28 | CC28 | 2 | UT | *mrp* ^+^ *epf* ^-^ *sly* ^-^ | AZM, CAM, CLDM, TC |
| 20SS43 | marketing | ST28 | CC28 | 2 | 2 | *mrp* ^+^ *epf* ^-^ *sly* ^-^ | AZM, CAM, CLDM, TC |
| 20SS44 | marketing | ST28 | CC28 | 2 | UT | *mrp* ^+^ *epf* ^-^ *sly* ^-^ | AZM, CAM, CLDM, TC |
| *S. suis* isolates from tonsils of pigs with endocarditis | | | | | | | |
| 20SS20 | marketing | ST28 | CC28 | 2 | 2 | *mrp* ^+^ *epf* ^-^ *sly* ^-^ | AZM, CAM, CLDM, TC |
| 20SS10 | marketing | ST117 (DLV of ST28) | CC28 | 3 | 3 | *mrp*** epf* ^-^ *sly* ^-^ | AZM, CAM, CLDM, TC |
| 20SS28 | marketing | ST1535 (DLV of ST795, ST1406) | NA | 15 | 15 | *mrp*** epf* ^-^ *sly* ^+^ | AZM, CAM, CLDM, TC |
| 20SS29 | marketing | ST1536 (DLV of ST1534, TLV of ST1683) | NA | 11 | UT | *mrp* epf * sly* ^+^ | AZM, CAM, CLDM, TC |
| 20SS30 | marketing | ST1537 | S | 31 | UT | *mrp* ^-^ *epf* ^-^ *sly* ^-^ | AZM, CAM, CLDM, TC, PCG |
| *S. suis* isolates from tonsils of healthy pigs | | | | | | | |
| 20SS04 | marketing | ST1526 (SLV of ST1) | CC1 | 2 | 2 | *mrp* ^-^ *epf* ^+^ *sly* ^+^ | AZM, CAM, CLDM, TC |
| 20SS07 | marketing | ST1526 (SLV of ST1) | CC1 | 2 | 2 | *mrp* ^-^ *epf* ^+^ *sly* ^+^ | AZM, CAM, CLDM, TC |
| 20SS05 | marketing | ST1526 (SLV of ST1) | CC1 | 2 | UT | *mrp* ^-^ *epf* ^+^ *sly* ^+^ | AZM, CAM, CLDM, TC |
| 20SS09 | marketing | ST13 | CC13 | 14 | UT | *mrp* ^-^ *epf* ^-^ *sly* ^+^ | No resistance |
| 20SS03 | marketing | ST17 | CC17 | 4 | 4 | *mrp* ^S^ *epf * sly* ^+^ | No resistance |
| 20SS08 | marketing | ST17 | CC17 | 4 | 4 | *mrp* ^S^ *epf * sly* ^+^ | No resistance |
| 20SS14 | marketing | ST17 | CC17 | 4 | 4 | *mrp* ^S^ *epf * sly* ^+^ | No resistance |
| 20SS18 | marketing | ST28 | CC28 | 2 | 2 | *mrp* ^+^ *epf* ^-^ *sly* ^-^ | No resistance |
| 20SS24 | marketing | ST28 | CC28 | 2 | 2 | *mrp* ^+^ *epf* ^-^ *sly* ^-^ | AZM, CAM, CLDM, TC |
| 20SS25 | marketing | ST28 | CC28 | 2 | 2 | *mrp* ^+^ *epf* ^-^ *sly* ^-^ | AZM, CAM, CLDM, TC |
| 21SS01 | marketing | ST28 | CC28 | 2 | 2 | *mrp* ^+^ *epf* ^-^ *sly* ^-^ | TC |
| 21SS17 | marketing | ST28 | CC28 | 2 | 2 | *mrp* ^+^ *epf* ^-^ *sly* ^-^ | TC |
| 21SS18 | marketing | ST28 | CC28 | 2 | 2 | *mrp* ^+^ *epf* ^-^ *sly* ^-^ | TC |
| 21SS21 | marketing | ST28 | CC28 | 2 | 2 | *mrp* ^+^ *epf* ^-^ *sly* ^-^ | AZM, CAM, CLDM, TC |
| 20SS06 | marketing | ST117 (DLV of ST28) | CC28 | 3 | 3 | *mrp*** epf* ^-^ *sly* ^-^ | AZM, CAM, CLDM, TC |
| 21SS04 | marketing | ST117 (DLV of ST28) | CC28 | 3 | 3 | *mrp*** epf* ^-^ *sly* ^-^ | AZM, CAM, CLDM, TC |
| 21SS07 | marketing | ST117 (DLV of ST28) | CC28 | 3 | 3 | *mrp*** epf* ^-^ *sly* ^-^ | AZM, CAM, CLDM, TC |
| 21SS16 | marketing | ST87 | CC87 | 8 | 8 | *mrp* ^-^ *epf* ^-^ *sly* ^+^ | No resistance |
| 20SS13 | marketing | ST1528 (DLV of ST87) | CC87 | 8 | 8 | *mrp* ^-^ *epf* ^-^ *sly* ^+^ | TC |
| 21SS10 | marketing | ST108 (SLV of ST94) | CC94 | 5 | 5 | *mrp* ^+^ *epf* ^-^ *sly* ^+^ | AZM, CAM, CLDM, TC |
| 20SS16 | marketing | ST1529 (TLV of ST94) | CC94 | 7 | 7 | *mrp* ^+^ *epf* ^-^ *sly* ^+^ | TC |
| 21SS09 | marketing | ST1679 (SLV of ST94) | CC94 | 4 | 4 | *mrp* ^+^ *epf* ^-^ *sly* ^+^ | AZM, CAM, CLDM, TC |
| 20SS15 | marketing | ST54 (SLV of ST53, ST1770, ST1805, ST1976, ST2093, ST2094, ST2097) | NA | 3 | 3 | *mrp* ^-^ *epf* ^-^ *sly* ^+^ | AZM, CAM, CLDM |
| 20SS11 | marketing | ST664 (DLV of ST163, TLV of ST172, ST192, ST477, ST718) | NA | 16 | 16 | *mrp* ^-^ *epf* ^-^ *sly* ^-^ | AZM, CAM, CLDM, TC |
| 20SS01 | marketing | ST1524 | S | 16 | 16 | *mrp* ^-^ *epf* ^-^ *sly* ^-^ | CLDM, TC |
| 20SS02 | marketing | ST1525 | S | UT | ND | *mrp* ^-^ *epf* ^-^ *sly* ^-^ | AZM, CAM, CLDM, TC |
| 20SS12 | marketing | ST1527 (DLV of ST802, TLV of ST138, ST650, ST807, ST1509) | NA | 9 | 9 | *mrp* ^-^ *epf* ^-^ *sly* ^-^ | AZM, CAM, CLDM, TC |
| 20SS19 | marketing | ST1530 | S | UT | ND | *mrp* ^-^ *epf* ^-^ *sly* ^-^ | AZM, CAM, CLDM, TC |
| 20SS21 | marketing | ST1531 (DLV of ST736, ST785) | NA | UT | ND | *mrp* epf* ^-^ *sly* ^+^ | TC |
| 20SS23 | marketing | ST1532 (DLV of ST1583, TLV of ST1914) | NA | 31 | 31 | *mrp* ^-^ *epf* ^-^ *sly* ^-^ | CLDM |
| 20SS26 | marketing | ST1533 (DLV of ST1800, ST2124, TLV of ST1161, ST1296, ST1300, ST1323, ST1864, ST1902) | NA | UT | ND | *mrp* ^-^ *epf* ^-^ *sly* ^+^ | AZM, CAM, CLDM, TC |
| 21SS05 | marketing | ST1533 (DLV of ST1800, ST2124, TLV of ST1161, ST1296, ST1300, ST1323, ST1864, ST1902) | NA | UT | ND | *mrp* ^-^ *epf* ^-^ *sly* ^+^ | AZM, CAM, CLDM, TC |
| 20SS27 | marketing | ST1534 (DLV of ST1536, TLV of ST1683) | NA | UT | ND | *mrp*** epf* ^-^ *sly* ^+^ | AZM, CAM, CLDM, TC |
| 20SS31 | marketing | ST1538 (TLV of ST661, ST1420) | NA | 16 | 16 | *mrp* ^-^ *epf* ^-^ *sly* ^-^ | No resistance |
| 20SS17 | marketing | ST1539 (DLV of ST38, ST751, TLV of ST231, ST639, ST641, ST857, ST2088) | NA | UT | ND | *mrp* ^-^ *epf * sly* ^+^ | CLDM |
| 21SS02 | marketing | ST1675 | S | 31 | 31 | *mrp* ^-^ *epf* ^-^ *sly* ^-^ | No resistance |
| 21SS03 | marketing | ST1676 | S | 5 | 5 | *mrp* ^-^ *epf* ^-^ *sly* ^-^ | No resistance |
| 21SS06 | marketing | ST1677 | S | 11 | 11 | *mrp* epf* ^-^ *sly* ^-^ | AZM, CAM, CLDM, TC |
| 21SS08 | marketing | ST1678 (SLV of ST1680) | NA | 12 | 12 | *mrp*** epf* ^-^ *sly* ^+^ | AZM, CAM, CLDM, TC |
| 21SS12 | marketing | ST1680 (SLV of ST1678) | NA | 12 | 12 | *mrp* epf* ^-^ *sly* ^+^ | AZM, CAM, CLDM, TC |
| 21SS13 | marketing | ST1681 | S | 31 | 31 | *mrp* ^-^ *epf* ^-^ *sly* ^-^ | CLDM, TC |
| 21SS14 | marketing | ST1682 (SLV of TS1683) | NA | UT | ND | *mrp* ^+^ *epf* ^-^ *sly* ^+^ | AZM, CAM, CLDM, TC |
| 21SS15 | marketing | ST1683 (SLV of TS1682, DLV of ST1534, ST1536) | NA | UT | ND | *mrp* epf* ^-^ *sly* ^+^ | AZM, CAM, CLDM, TC |
| 21SS19 | marketing | ST1684 (TLV of ST1342) | NA | 31 | 31 | *mrp* ^-^ *epf* ^-^ *sly* ^-^ | CLDM, TC, PCG |
| 21SS20 | marketing | ST1685 (TLV of ST1000) | NA | 10 | 10 | *mrp* ^-^ *epf* ^-^ *sly* ^-^ | AZM, CAM, CLDM, TC |

SLV, single-locus variant. DLV, double-locus variant. TLV, Triple-locus variant. CC, clonal complex. NA, no clonal complex could be assigned and was a single- to triple-locus variant of a certain ST. S, singleton, i.e., more than 4 loci were different from other STs. UT, untypeable, i.e., *cps* type could not be determined or bacteria were not agglutinated by the expected antiserum. ND, not done. Ages of pigs are as follows; marketing (approximately 6 months old), reproductive (approximately 2 to 3 years old). Variants of MRP are shown as *mrp*^S^ (747 bp), *mrp* (1148 bp), *mrp** (1556 bp), and *mrp**** (2400 bp). *epf**, a large-size variant of *epf*.
