# Supplementary material for: Characterization of pig tonsils as niches for the generation of Streptococcus suis diversity
Source: Vet Res. 2024 Feb 6;55:17. doi: 10.1186/s13567-024-01270-5 (PMC10848530; doi:10.1186/s13567-024-01270-5)
Supplement: Supplementary file 2 — Additional file 2. Eight novel sequence types (STs) assigned to singletons and their relationships to other STs. [file 13567_2024_1270_MOESM2_ESM.docx]

**Additional file 2. Eight novel sequence types (STs) assigned to singletons and their relationships to other STs.**

| ST | Relationship |
| --- | --- |
| ST1524 | Quadruple-locus variant of ST477, ST664a, ST672, ST723, and ST1933.  Quintuple-locus variant of ST41, ST56, ST58, ST163, ST172, ST191, ST192, ST281, ST300, ST718, ST795, ST870, ST877, ST884, ST892, ST916, ST1037, ST1301, ST1367, ST1457, ST1535a, ST1904, ST1991, and ST2173a. |
| ST1525 | Quintuple-locus variant of ST797 and ST1319. |
| ST1530 | Quintuple-locus variant of ST1401, ST2032, and ST2044. |
| ST1537 | Quadruple-locus variant of ST631.  Quintuple-locus variant of ST75, ST163a, ST209, ST269, ST624, ST682, ST892, ST991, ST995, ST1129, ST1179, ST1215, ST1222, ST1638, ST1677, ST1700, ST1874, ST1884, and ST1912. |
| ST1675 | Quadruple-locus variant of ST950.  Quintuple-locus variant of ST109a, ST876, and ST1676a. |
| ST1676 | Quintuple-locus variant of ST364, ST1009a, and ST1675a. |
| ST1677 | Quintuple-locus variant of ST106, ST163a, ST218, ST628, ST661, ST730, ST789, ST865, ST866, ST995, ST1420, ST1421, ST1537, ST1538, ST1827, ST1889, and ST1995. |
| ST1681 | Quadruple-locus variant of ST139.  Quintuple-locus variant of ST210 and ST249. |

a STs that appear in Figure 3
