# Supplementary material for: Characterization of pig tonsils as niches for the generation of Streptococcus suis diversity
Source: Vet Res. 2024 Feb 6;55:17. doi: 10.1186/s13567-024-01270-5 (PMC10848530; doi:10.1186/s13567-024-01270-5)
Supplement: Supplementary file 3 — Additional file 3. STs that were Single-locus Variants (SLVs) or Double-locus variants (DLVs) of other STs originating from different countries. [file 13567_2024_1270_MOESM3_ESM.docx]

**Additional file 3. STs that were Single-locus Variants (SLVs) or Double-locus variants (DLVs) of other STs originating from different countries.**

| ST (Country) | Linking level and STs (Country) |
| --- | --- |
| ST17 (Japan) | SLV of ST1011 (Spain) and ST2224 (China).  DLV of ST2235 (China), ST1010 (Spain), and ST2157 (the Philippines). |
| ST28 (Japan, Vietnam) | SLV of ST205(Vietnam), ST287 (China) and ST1432(Canada).  DLV of ST299(Thailand), ST1648(China). |
| ST87 (Japan) | DLV of ST2246 (Canada) |
| ST94 (Japan, Thailand) | SLV of ST108 (Japan), ST1679 (Japan), and ST2220 (China). |
| ST115 (Japan) | DLV of ST1913 (China). |
| ST664 (Japan) | SLV of ST163 (China). |
| ST802 (the Netherlands) | DLV of ST1527 (Japan). |
| ST1526 (Japan) | SLV of ST1 (Thailand, Vietnam, Germany, United Kingdom) |
| ST1583 (China) | DLV of ST1532 (Japan). |

STs that appear in Figure 3. All isolates belonging to these STs were obtained from tonsils.
